# The direction of clinical pharmacy education in Japan

**DOI:** 10.5116/ijme.618e.26f9

**Published:** 2021-12-07

**Authors:** Takanao Hashimoto, Naho Sasaki, Fumiyoshi Ojima

**Affiliations:** 1 Department of Pharmacy, Sendai City Medical Center, Japan; 2 Tsubasa Pharmacy, Japan; 3 Clinical Pharmacy Practice Center, Tohoku Medical and Pharmaceutical University, Japan

**Keywords:** Learning objectives, model core curriculum, pharmacy education, Japan

## To the Editor

In Japan, pharmacy education shifted from a four-year to a six-year program in 2006, and the Model Core Curriculum was established to raise the level of pharmacy education. This curriculum requires pharmacy students to learn about eight representative diseases (cancer, hypertension, diabetes, cardiovascular diseases, cerebrovascular diseases, neuropsychiatric diseases, immunological and allergic diseases, and infectious diseases) during clinical practice training in community pharmacies and hospitals.^1^ The duration of practical training at a pharmacy and a hospital are 11 weeks each. In this report, we discuss the direction of clinical pharmacy education in Japan.

As part of its pre-training program, since 2017, the Faculty of Pharmacy, Tohoku Medical and Pharmaceutical University (TMPU) has provided interprofessional education and collaborated with TMPU Hospital, a nursing college, and a university to train various healthcare professionals.^2^ Through simulated cases, students are able to gain knowledge of patient-centered medicine as part of a multidisciplinary medical team.

Herein, we introduce an example in which a pharmacy student contributed to improving the quality of life of a patient in the home care setting through community pharmacy training. An elderly patient with dementia and diabetes mellitus that required insulin was using candy to prevent hypoglycemia. However, some of the candies were sugarless. The student noticed this and made a candy container that separated the candies with sugar from the sugarless ones at the next visit. This patient remembered it even after the student's clinical training had finished and continued to use the candy container.

In-hospital training, we provided pharmacy students with the opportunity to learn the skill of providing drug information (DI).^3^ Pharmacy students studied the assessment of adverse drug events (ADEs) and wrote ADE reports for the Pharmaceuticals and Medical Devices Agency (PMDA) in Japan. The PMDA is an administrative organization dealing with ADEs and the relief system in Japan. These ADE reports included Stevens-Johnson Syndrome caused by Ramusirumab and Docetaxel combination therapy for non-small cell lung carcinoma and anaphylaxis by an iodinated contrast agent, both of which were clinical cases of Sendai City Medical Center.

In addition, representative pharmacy students of the TMPU participated in a health event for Sendai citizens organized by the Sendai City Pharmacists Association.^4^ In this event, participants, most of whom were elderly, learned about the appropriate use of generic medicine, prevention of drug abuse, and how to maintain their health. The pharmacy students joined the event as staff members.

We had conducted a DI workshop for pharmacy students at the Pharmacy Student Festival 2019 organized by The Association of Pharmaceutical Students – Japan.^5^ In this workshop, we provided two clinical cases. In the first case, a patient had stopped taking amlodipine to prevent potential side effects, as he believed that amlodipine would lead to rhabdomyolysis (an ADE that was reported in spontaneous reports). The pharmacy students examined the limitations of spontaneous ADE reports and discussed ways to facilitate the patient's understanding. In the second case, a patient suffered from intestinal perforation caused by oral barium sulfate during a medical checkup, but he believed that it may have been a medication error. The pharmacy students studied the relief systems for ADEs in Japan by referring to the website of the PMDA and discussed the difference between medication errors and relief cases.

The current situation of pharmacy education in Japan has the following challenges. First, the current practical pharmacy training in Japan does not cover all "eight representative diseases" that pharmacy students need to learn. We found that the percentage of TMPU students who had the opportunity to learn all eight representative diseases in 2019 was approximately 9% (Unpublished data). Second, despite the rapid changes in Japan's healthcare setting because of its super-aged society, pharmacy education may not be able to adequately respond to the changes. There have not been enough opportunities for pharmacy students to learn advanced care planning and end-of-life care.

We introduce representative cases and the challenges of clinical pharmacy education in Japan. To improve the quality of pharmacy education, continuous communication with other healthcare schools is needed.

### Conflict of Interest

The authors declare that they have no conflict of interest.

## References

[1] Council on pharmaceutical education. [Cited 3 Nov 2020]; Available from: https://yaku-kyou.org/.

[2] Tohoku Medical and Pharmaceutical University, Interprofessional education. [Cited 2 Feb 2021]; Available from: https://www.tohoku-mpu.ac.jp/about/information/cooperation/.

[3] Hashimoto T, Honmei T, Tochikubo K. Practical drug information training for higher learning effect - drug information news, adverse drug reaction reporting, and report for debriefing session. Journal of Japanese Society of Hospital Pharmacists. 2020;56(12):1412-8.

[4] The Sendai City Pharmacists Association. The health event for citizens. [Cited 3 Oct 2021]; Available from: https://senyaku.org/?p=830.

[5] Japan Pharmacy Students' Festival 2019. [Cited 24 Dec 2020]; Available from: https://ynps.yakuji.co.jp/8609.html.

